# Region-specific deep learning models for accurate segmentation of rectal structures on post-chemoradiation T2w MRI: a multi-institutional, multi-reader study

**DOI:** 10.3389/fmed.2023.1149056

**Published:** 2023-05-11

**Authors:** Thomas DeSilvio, Jacob T. Antunes, Kaustav Bera, Prathyush Chirra, Hoa Le, David Liska, Sharon L. Stein, Eric Marderstein, William Hall, Rajmohan Paspulati, Jayakrishna Gollamudi, Andrei S. Purysko, Satish E. Viswanath

**Affiliations:** ^1^Department of Biomedical Engineering, Case Western Reserve University, Cleveland, OH, United States; ^2^Department of Colorectal Surgery, Cleveland Clinic, Cleveland, OH, United States; ^3^Department of Surgery, University Hospitals Cleveland Medical Center, Cleveland, OH, United States; ^4^Northeast Ohio Veterans Affairs Medical Center, Cleveland, OH, United States; ^5^Department of Radiation Oncology and Surgery, Medical College of Wisconsin, Milwaukee, WI, United States; ^6^Department of Diagnostic Imaging and Interventional Radiology, Moffitt Cancer Center, Tampa, FL, United States; ^7^Department of Radiology, University of Cincinnati, Cincinnati, OH, United States; ^8^Section of Abdominal Imaging and Nuclear Radiology Department, Cleveland Clinic, Cleveland, OH, United States

**Keywords:** rectal cancer (RC), deep learning, MRI, segmentation, U-Net, artificial intelligence (AI)

## Abstract

**Introduction:**

For locally advanced rectal cancers, *in vivo* radiological evaluation of tumor extent and regression after neoadjuvant therapy involves implicit visual identification of rectal structures on magnetic resonance imaging (MRI). Additionally, newer image-based, computational approaches (e.g., radiomics) require more detailed and precise annotations of regions such as the outer rectal wall, lumen, and perirectal fat. Manual annotations of these regions, however, are highly laborious and time-consuming as well as subject to inter-reader variability due to tissue boundaries being obscured by treatment-related changes (e.g., fibrosis, edema).

**Methods:**

This study presents the application of U-Net deep learning models that have been uniquely developed with region-specific context to automatically segment each of the outer rectal wall, lumen, and perirectal fat regions on post-treatment, T_2_-weighted MRI scans.

**Results:**

In multi-institutional evaluation, region-specific U-Nets (wall Dice = 0.920, lumen Dice = 0.895) were found to perform comparably to multiple readers (wall inter-reader Dice = 0.946, lumen inter-reader Dice = 0.873). Additionally, when compared to a multi-class U-Net, region-specific U-Nets yielded an average 20% improvement in Dice scores for segmenting each of the wall, lumen, and fat; even when tested on T_2_-weighted MRI scans that exhibited poorer image quality, or from a different plane, or were accrued from an external institution.

**Discussion:**

Developing deep learning segmentation models with region-specific context may thus enable highly accurate, detailed annotations for multiple rectal structures on post-chemoradiation T_2_-weighted MRI scans, which is critical for improving evaluation of tumor extent *in vivo* and building accurate image-based analytic tools for rectal cancers.

## 1. Introduction

Colorectal cancer is the third most diagnosed cancer worldwide, with nearly 40% of tumors localized to the rectum (1). Patients diagnosed with locally advanced rectal cancer (LARC) typically undergo neoadjuvant chemoradiation (nCRT) (2), after which Magnetic Resonance Imaging (MRI) is routinely acquired for evaluation of treatment response *in vivo* in order to guide follow-up patient management (3, 4). Radiological evaluation of post-nCRT MRI scans typically involves implicit visual identification of rectal structures on MRI (namely the lumen, outer rectal wall, and perirectal fat), where the boundaries can be obscured as a result of treatment effects including fibrosis, edema, or necrosis (5). This limits the sensitivity of radiological evaluation in determining tumor extent and regression *in vivo* (e.g., tumor stage or T-stage) (6, 7). Additionally, newer computational radiomics approaches (the computerized extraction of quantitative measurements from radiographic imaging) require precise delineation of the lumen, rectal wall, and perirectal fat (8, 9) on MRI. The latter task is not only highly manual and labor intensive but can be complicated by the confounded boundaries of rectal structures on post-nCRT MRI. There is thus an unmet clinical need for specialized models that can identify rectal structures on post-nCRT MRI to enable more detailed characterization of tumor impact as well as build accurate downstream analytical pipelines.

Deep learning models, such as fully convolutional neural networks (FCNs), have recently shown wide-ranging success in medical imaging segmentation tasks (10), especially using the popular U-Net architecture (11). Previously presented segmentation approaches in the context of rectal MRI scans, summarized in Supplementary Table 1, have primarily focused on delineation of tumor alone while comparing against a single reader annotation on pre-nCRT MRI cohorts (12–17). However, FCNs trained on pre-nCRT MRI scans may not be optimized for delineating regions of interest (ROIs) on post-nCRT MRI scans, as the rectal environment on post-nCRT MRI is visually and pathologically distinct from pre-nCRT MRI (6, 18). Previous studies most directly related to the current work have leveraged U-Nets to automatically segment both tumor and rectal wall (14) as well as to delineate tumor, rectal wall, and the perirectal fat; albeit using only single reader annotations on data from a single institution (17). Critically, the utility of such segmentation models is contingent on their performance across multiple institutions to confirm their generalizability among a variety of imaging settings and differences in image quality. In order to reduce the burden of expert annotation, it is also important for these models to be comparable to the agreement between multiple radiology readers.

Research in other tumor types has also suggested region-specific models may be more effective and accurate in identifying region boundaries than a traditional multi-class model (19, 20). Thus, the traditional multi-class segmentation approach to simultaneously delineate multiple ROIs utilized in previous studies may be sub-optimal for identifying boundaries of different structures in the rectal environment on post-nCRT MRI. The hypothesis of this study is that U-Nets trained with region-specific context could enable more accurate, automated delineation of different rectal structures (the outer rectal wall, lumen, and perirectal fat) on post-nCRT MRI.

The contributions of this work are threefold:

1. Initial results of developing U-Net models trained with region-specific context to automatically identify boundaries of the outer rectal wall, lumen, and perirectal fat on post-nCRT T_2_-weighted MRI scans.
2. Performance evaluation of region-specific U-Nets in a multi-reader setting, against repeat annotations from two radiology readers.
3. Validation of region-specific U-Nets performance across multiple imaging settings and institutional cohorts.
  Per the current understanding of the field, this is one of the first efforts in developing deep learning models specifically optimized for post-nCRT MRI, as well as for accurately delineating multiple rectal structures in detail.

## 2. Materials and methods

Here, U-Net models are developed with region-specific context to accurately delineate rectal structures on post-nCRT MRI. A total of 92 patients were deemed eligible for this study. All MRIs underwent pre-processing to account for variance in imaging acquisition across all institutions. Subsequently, three U-Net models were trained to delineate the outer rectal wall, lumen, and perirectal fat on a 2D basis. The output segmentations of the region-specific U-Net models were compared to a traditional multiclass U-Net model. The following sections describe specific details of preprocessing steps, model parameters, experimental design, and evaluation of model performance.

### 2.1. Patient selection and dataset description

**FIGURE 1:**
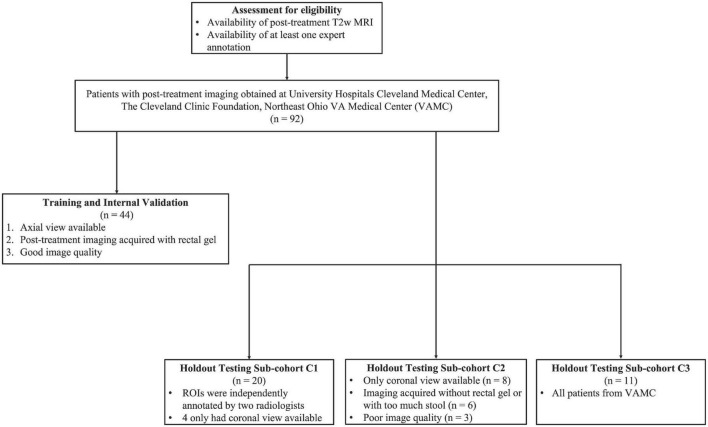
Flow diagram of patient eligibility and exclusion criteria of the multi-institutional dataset used in this study.

In this IRB-approved study, patients diagnosed and treated for rectal adenocarcinoma between August 2007 and October 2015 were curated from three different institutions [University Hospitals Cleveland Medical Center (UHCMC), Cleveland Clinic (CC), and Northeast Ohio VA Medical Center (NOVAMC)]. In total, 92 patients were deemed eligible for this study based on the availability of at least one post-nCRT T_2_-weighted (T2w) MRI, with the entirety of the outer rectal wall, lumen, and perirectal fat annotated by at least one radiologist. Due to the retrospective nature of this study, informed consent was waived, as all data was de-identified prior to experimental analysis. Figure 1 illustrates the inclusion/exclusion criteria employed to split the 92 patients into training and holdout testing cohorts. The training and internal validation cohort comprised 44 patients from UHCMC and CC based on three criteria: (i) availability of axial T2w MRI, (ii) administration of rectal gel when preparing the patient for MR imaging, and (iii) determined to be high-quality upon visual inspection (no motion artifacts, high contrast differences between rectal structures).

The remaining 48 patients were placed into the holdout testing cohort, which was further divided into three sub-cohorts (Figure 1). Sub-cohort C1 comprised 20 patients with repeat annotations of outer rectal wall, lumen, and perirectal fat regions which had been done independently by two radiologists with 13 and 7 years of experience, respectively, to determine whether U-Net models trained with region-specific context were comparable to multiple readers, as well as their inter-reader agreement. To further test the robustness of the region-specific U-Net models, 17 patients whose imaging characteristics were different from training inclusion criteria (e.g., coronal MRI, without rectal gel, or visibly poor image quality) were curated to form sub-cohort C2. Finally, 11 T2w MRI scans from NOVAMC were included in sub-cohort C3, representing an external validation set (i.e., an institution not included in the training cohort).

### 2.2. MRI acquisition characteristics

All included patients had been imaged after nCRT for locally advanced disease, using a T_2_-weighted turbo spin echo sequence at each institution. Across all three institutions, T_2_-weighted MRI was acquired via two different scanner manufacturers (Philips and Siemens) and 10 unique models, resulting in varied imaging parameters (in-plane resolution: 0.313–1.172 mm, slice thickness: 3.0–8.0 mm, repetition time: 2400–11800 ms, echo time: 64–184 ms). However, imaging parameters within each institution were fairly consistent.

### 2.3. Annotation of the outer rectal wall, lumen, and perirectal fat on post-nCRT T2w MRI

Using available clinical, pathologic, and radiology reports, as well as all imaging planes and any additional imaging sequences, a radiologist at each institution manually annotated the entirety of the outer rectal wall, lumen, perirectal fat, and obturator internus muscle on each post-nCRT T2w MRI dataset. For datasets in C1, two radiologists independently annotated the outer rectal wall, lumen, and perirectal fat in a blinded fashion. All annotations were performed in 3D Slicer (21). Sigmoidal colon regions above the peritoneal reflection, as well as regions below the top of the anal canal, were omitted for annotation purposes.

### 2.4. Processing of MRI scans for acquisition differences and artifacts

**FIGURE 2:**
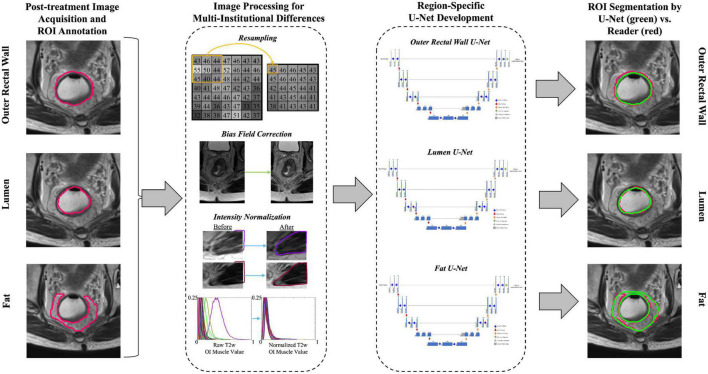
Overview of study workflow for developing region-specific U-Nets for segmentation of the outer rectal wall, lumen, and perirectal fat on post-treatment, T2w MRI. Image processing included resampling to a common resolution, bias field correction, and intensity normalization.

An overview of the experimental workflow (including all processing steps) is presented in Figure 2. To correct for resolution differences across the three institutions, all volumes were linearly re-sampled to a common resolution (0.781 mm × 0.781 mm × 4 mm, selected as the most commonly occurring resolution across all included patients). Next, inhomogeneity in gray-level intensities resulting from bias field was addressed via the N4ITK bias field correction algorithm (22). Finally, T2w signal intensities within the outer rectal wall, lumen, and perirectal fat were normalized with respect to the mean intensity of the obturator internus muscle within each MRI dataset, to account for marked intensity variations across all three institutions.

### 2.5. Region-specific and multiclass U-Net segmentation architecture

The U-Net FCN architecture (11) was implemented for segmentation of different rectal structures due to its wide popularity for biomedical image segmentation. Within the U-Net, the contractive path extracts features from images via convolution blocks like a typical convolutional neural network. Each convolution block contained two sequences of 3 × 3 convolution followed by batch normalization. The output of each block was downsampled by a 2 × 2 max pooling operation. The expansive path then uses spatial information from the contractive path via a series of up-convolutions and skip connections to ultimately produce a pixel-wise segmentation of the original input image. In the expansive path, each convolution block contained a 3 × 3 up-convolution, concatenation with the corresponding feature map from the contractive path, and two sequences of 3 × 3 convolution (followed by batch normalization). Non-linearity was introduced into each layer of convolution in the contractive and expansive paths via ReLu (Rectified Linear Units), except for the output layer which used a sigmoid activation function to produce binary segmentations.

Three separate region-specific U-Nets were developed to identify the boundaries of different rectal structures on MRI: (i) the outer rectal wall boundary (denoted B*^W^*), (ii) the outer lumen boundary (denoted B*^L^*, which is also the inner rectal wall boundary), and (iii) the perirectal fat (denoted B*^F^*). A fourth multi-class U-Net was additionally developed to segment B*^W^*, B*^L^*, and B*^F^* simultaneously.

### 2.6. Experimental design

The three region-specific U-Nets as well as the multiclass U-Net shared identical architectures and hyperparameters, except for the activation function in the final output layer. These parameters were empirically determined via a gridsearch optimization strategy. The multiclass U-Net used a SoftMax activation function (as each pixel was assigned a probability of belonging to each region), whereas the region-specific U-Nets utilized a sigmoid activation function to produce binary segmentations for each region. All U-Nets were trained over 50 epochs, with a batch size of 16, the Adam optimization function (learning rate = 0.003) (23), and a Dice Similarity Coefficient (DSC) loss function. Dropout regularization of 0.2 was implemented to prevent overfitting (24). Images were center-cropped to remove extraneous information (e.g., hip bones, bladder) and resized to 128 × 128 before being input to the networks. Images were then augmented on-the-fly with vertical flips and rotations between −30 and 30° to improve generalizability. The U-Net networks were implemented in Keras, using a Tensorflow backend (25), and trained via 2 NVIDIA Tesla P100 GPUs with a total of 16 GB of memory at the high performance computer cluster of Case Western Reserve University. Following training, the threshold to binarize the segmentation maps generated by each U-Net was optimized on the internal validation set. The binary segmentations for each region were further refined via connected component analysis (CCA) (26) to retain only the largest connected component (assumed to be the primary region being segmented in each case).

### 2.7. Evaluation of U-Net models and statistical analysis

Three different measures were utilized to evaluate all segmentation results:

• Dice similarity coefficient (DSC) was used to quantify the overlap between two segmentations as follows:

$$D\,S\,C\,\left(X,Y\right)=\frac{2\,\left|X\,⋂Y\right|}{\left|X\right|+\left|Y\right|}$$

where X is the U-Net segmentation and Y is the expert annotation. DSC values lie between 0 and 1, where DSC = 1 indicates a perfect overlap.

• Hausdorff distance (HD) was calculated as the maximum Euclidean distance between every point along two boundaries:

$$H\,D\,\left(X,Y\right)=m\,a\,x\,\left\{s\,u\,p_{a\in X}\,i\,n\,f_{b\in Y}\,d\,\left(a-b\right),s\,u\,p_{b\in Y}\,i\,n\,f_{a\in X}\,d\,\left(a-b\right)\right\}$$

where *d(a-b)* is the Euclidean distance between points *a* and *b* that belong to the U-Net segmentation, *X*, and the expert annotation, *Y*, respectively (27). A HD of 0 indicates that the two contours are identical, and thus the higher the HD, the more different the two boundaries are from each other.

• Fréchet distance (FD) was used to quantify the Euclidean distance between two boundaries while also taking the continuity of the contours into account, and is defined as follows:

$$FD\left(X,Y\right)=inf_{\alpha ,\beta }max_{t\in \left[0,1\right]}\left\{d\left(X\left(\alpha \right)\left(t\right)\right),Y\left(\beta \left(t\right)\right)\right)\}$$

where α and β are parameterizations of the U-Net segmentation, *X*, and the expert annotation, *Y*, respectively, and *d* is the Euclidean distance between them (28). An FD of 0 corresponds to the two contours being identical, whereas a higher FD indicates more dissimilarity between them.

The performance of the region-specific U-Nets and multiclass U-Net were evaluated against annotations of B*^W^*, B*^L^*, and B*^F^* by each of the two readers, as well as the inter-reader agreement, in holdout testing sub-cohort C1, via median DSC, HD, and FD. Statistically significant differences in performance between the different U-Net models and inter-reader agreement were assessed via a pairwise Wilcoxon ranksum test, using Bonferroni’s correction to account for multiple comparisons. In sub-cohort C2, region-specific U-Net and multiclass U-Net delineations of B*^W^*, B*^L^*, and B*^F^* were evaluated against a single reader’s annotations in terms of median DSC, HD, and FD within subgroups based on differing imaging characteristics (grouped by coronal view images, images acquired without rectal gel, and images of poor quality). External evaluation of region-specific U-Net and multiclass U-Net segmentation performances was conducted in sub-cohort C3 (from a third institution) also via median DSC, HD, and FD against a single set of reader annotations. For both C2 and C3, Wilcoxon testing was used to determine significant differences in performance (if any) between the region-specific U-Nets and the multiclass U-Net.

## 3. Results

### 3.1. Experiment 1: performance of region-specific U-Net delineations compared to inter-reader agreement

**FIGURE 3:**
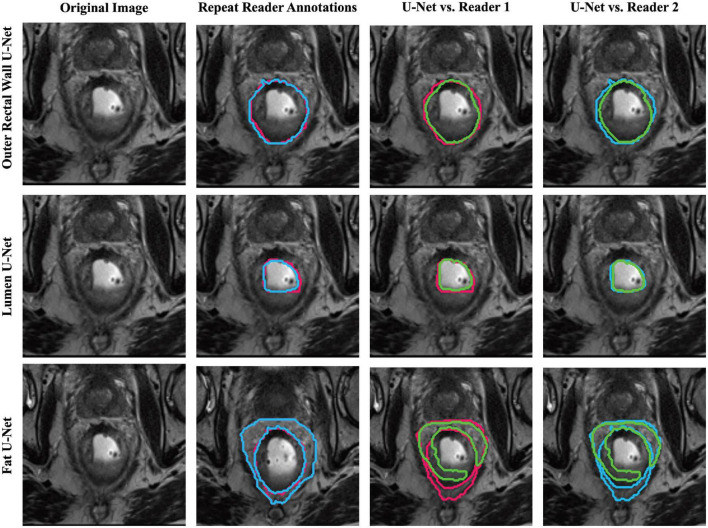
Representative segmentations of each region-specific U-Net (green) compared to annotations of reader 1 (red) and reader 2 (blue) on a single patient in holdout testing subcohort C1.

Table 1 summarizes the performance of region-specific U-Net delineations of B*^W^*, B*^L^*, and B*^F^* when evaluated against annotations from each of reader 1 and reader 2 on the holdout testing sub-cohort C1. Of the three region-specific U-Nets, the B*^W^* U-Net achieved the best segmentation performance compared to either reader with a median DSC > 0.9 as well as median HD and FD < 2.85 mm. The B*^F^* U-Net yielded moderate segmentation performance compared to either reader, with an overall median DSC around 0.7 as well as median HD and FD > 3.85 mm. In general, region-specific B*^W^* and B*^L^* U-Nets yielded DSC, FD, and HD values that were not significantly different from inter-reader agreement (*p* > 0.008). Figure 3 further illustrates the relatively high similarity in boundary delineations achieved for each of B*^W^*, B*^L^*, and B*^F^* via region-specific U-Nets compared to each set of reader annotations, on representative 2D MR images from holdout testing sub-cohort C1. Supplementary Table 2 presents quantitative results for the performance of the multi-class U-Net delineations of B*^W^*, B*^L^*, and B*^F^* when evaluated against annotations from each of reader 1 and reader 2 on holdout testing sub-cohort C1; which demonstrate that (i) region-specific U-Nets outperformed the multiclass U-Net in all comparisons, and (ii) the multiclass U-Net yielded significantly worse DSC, HD, and FD compared to the inter-reader agreement (*p* < 0.008). Supplementary Figure 1 visualizes representative multiclass U-Net segmentations of outer rectal wall (top row), lumen (middle row), and fat (bottom row), in comparison to annotations from each of reader 1 and reader 2.

**TABLE 1:** Inter-reader agreement (reader 1 vs. reader 2) and performance of each region-specific U-Net on holdout testing sub-cohort C1.

Region	Comparison	Median dice	Median Hausdorff distance	Median Fréchet distance
Outer rectal wall	Reader 1 vs. Reader 2	0.946 ± 0.042	2.65 ± 0.584	2.83 ± 0.645
	B^W^ U-Net vs. Reader 1	0.920 ± 0.060*	2.65 ± 0.621	2.83 ± 0.690
	B^W^ U-Net vs. Reader 2	0.924 ± 0.066*	2.65 ± 0.614	2.83 ± 0.732
Lumen	Reader 1 vs. Reader 2	0.873 ± 0.199	2.45 ± 0.601	2.83 ± 0.613
	B^L^ U-Net vs. Reader 1	0.895 ± 0.215	2.45 ± 0.733	2.65 ± 0.731
	B^L^ U-Net vs. Reader 2	0.898 ± 0.181	2.45 ± 0.656	2.45 ± 0.708
Fat	Reader 1 vs. Reader 2	0.866 ± 0.261	3.16 ± 0.847	3.46 ± 0.947
	B^F^ U-Net vs. Reader 1	0.696 ± 0.360*	3.87 ± 1.06*	4.36 ± 1.11*
	B^F^ U-Net vs. Reader 2	0.729 ± 0.359*	3.87 ± 1.04*	4.24 ± 1.08*

*Denotes *p*-values < 0.008. *p*-values were calculated by Wilcoxon Ranksum.

### 3.2. Experiment 2: performance of region-specific U-Nets on validation cohort with differing imaging characteristics

**FIGURE 4:**
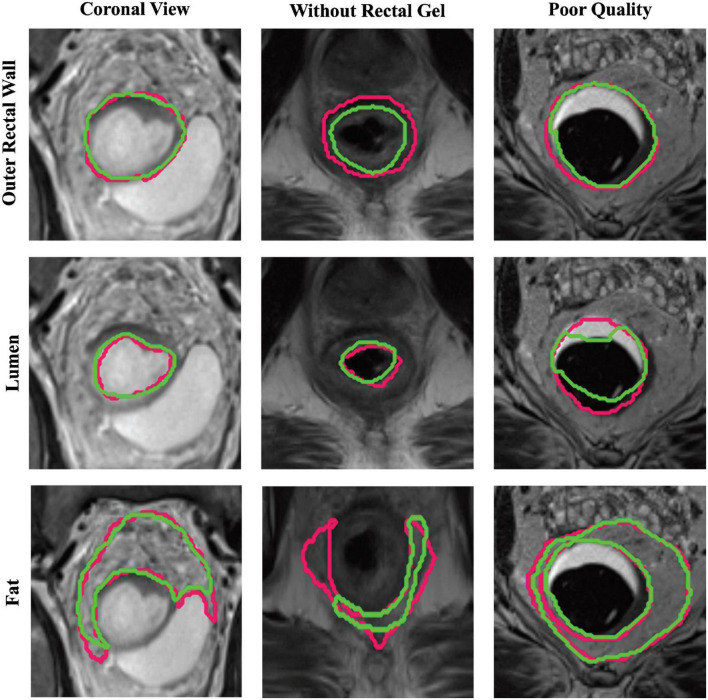
Representative segmentations via region-specific U-Nets (green) compared to expert annotations (pink) on patients from each sub-group of holdout testing cohort C2. Top row, middle row, and bottom row illustrate BW, BL, and BF U-Net segmentations, respectively, for a scan in the coronal view **(first column)**, scan without rectal gel **(second column)**, and a scan of visibly poor quality **(last column)**.

For each group of patients in sub-cohort C2 (coronal view, no rectal gel, poor image quality), the performances of different U-Net strategies are summarized in Table 2, demonstrating that region-specific U-Nets yield significantly higher DSC, HD, and FD for each of B*^W^* and B*^L^* compared to the multiclass U-Net (*p* < 0.008). Figure 4 illustrates the relatively accurate boundary delineation for B*^W^* and B*^L^* region-specific U-Nets compared to reader annotations for representative images from all three image groups. By comparison, the region-specific B*^F^* U-Net is seen to vary in performance between image groups in sub-cohort C2, with marginally better performance in cases acquired in the coronal plane or those acquired without rectal gel compared to scans of poor image quality.

**TABLE 2:** Performance of region-specific U-Nets and multiclass U-Net on holdout testing sub-cohort C2.

Cohort	Region	U-Net	Median dice	Median Hausdorff distance	Median Fréchet distance
Excluded—Coronal view	Outer rectal wall	B^W^ U-Net	0.903 ± 0.150*	2.65 ± 0.823*	2.83 ± 0.820*
		Multiclass	0.756 ± 0.162*	3.46 ± 0.799*	3.74 ± 0.830*
	Lumen	B^L^ U-Net	0.833 ± 0.167*	2.65 ± 0.622*	2.65 ± 0.577*
		Multiclass	0.575 ± 0.263*	3.32 ± 0.819*	3.46 ± 0.808*
	Fat	B^F^ U-Net	0.377 ± 0.376	5.00 ± 1.37	5.10 ± 1.29
		Multiclass	0.408 ± 0.386	4.47 ± 1.32	4.58 ± 1.25
Excluded—No rectal gel	Outer rectal wall	B^W^ U-Net	0.877 ± 0.136*	3.00 ± 0.759*	3.16 ± 0.767*
		Multiclass	0.763 ± 0.176*	3.32 ± 0.902*	3.46 ± 0.887*
	Lumen	B^L^ U-Net	0.775 ± 0.222*	3.00 ± 0.833	3.16 ± 0.862
		Multiclass	0.649 ± 0.267*	3.00 ± 0.874	3.32 ± 0.898
	Fat	B^F^ U-Net	0.500 ± 0.354	4.00 ± 1.49	4.18 ± 1.13
		Multiclass	0.497 ± 0.364	3.87 ± 1.06	4.12 ± 1.07
Excluded—Poor quality	Outer rectal wall	B^W^ U-Net	0.827 ± 0.167*	3.16 ± 0.838*	3.53 ± 0.864*
		Multiclass	0.571 ± 0.301*	4.69 ± 1.23*	4.90 ± 1.14*
	Lumen	B^L^ U-Net	0.678 ± 0.163*	2.91 ± 0.805	3.16 ± 0.914
		Multiclass	0.324 ± 0.362*	3.53 ± 0.548	3.53 ± 0.471
	Fat	B^F^ U-Net	0.454 ± 0.224	4.36 ± 0.983	5.15 ± 1.01
		Multiclass	0.451 ± 0.281	4.24 ± 1.00	5.00 ± 1.18

*Denotes *p*-values < 0.008. *p*-values were calculated by Wilcoxon Ranksum.

### 3.3. Experiment 3: performance of region-specific U-Nets on external validation cohort from a different institution

**FIGURE 5:**
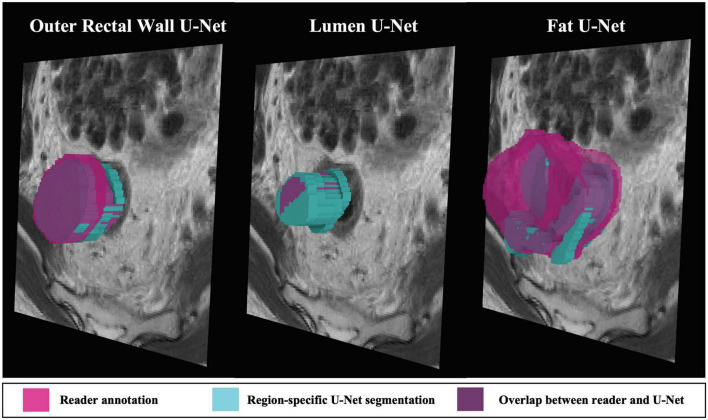
3D representations of region-specific segmentations on holdout testing sub-cohort C3. Reader annotations denoted in pink, while cyan denotes BW, BL, and BF U-Net segmentations. Purple indicates overlap between reader annotation and U-Net delineation.

On the external validation sub-cohort C3, region-specific B*^W^*, B*^L^*, and B*^F^* U-Nets achieved relatively high performance in terms of DSC, HD, and FD (summarized in Table 3), which were also significantly higher than the multiclass U-Net for B^W^ and B^L^ (*p* < 0.008). Figure 5 depicts representative 3D visualizations of region-specific boundary delineations (cyan) together with corresponding reader annotations (pink), for each of the rectal wall, lumen, and perirectal fat. Note the predominance of overlapping regions (shaded in purple) between reader annotations and B^W^ and B^F^ U-Net delineations, compared to slight over-segmentation achieved by B^L^ U-Net (higher proportion of blue).

**TABLE 3:** Performance of region-specific U-Nets and multiclass U-Net holdout testing sub-cohort C3.

Cohort	Region	U-Net	Median dice	Median Hausdorff distance	Median Fréchet distance
VA	Outer rectal wall	B^W^ U-Net	0.913 ± 0.056*	2.45 ± 0.516*	2.45 ± 0.557*
		Multiclass	0.604 ± 0.233*	3.46 ± 0.845*	3.53 ± 0.841*
	Lumen	B^L^ U-Net	0.804 ± 0.154*	2.45 ± 0.690*	2.65 ± 0.701*
		Multiclass	0.614 ± 0.280*	3.00 ± 0.903*	3.00 ± 0.888*
	Fat	B^F^ U-Net	0.791 ± 0.284	4.00 ± 0.821	4.58 ± 0.862
		Multiclass	0.807 ± 0.286	3.87 ± 0.815	4.58 ± 0.820

*Denotes *p*-values < 0.008. *p*-values were calculated by Wilcoxon Ranksum.

## 4. Discussion

In this multi-institutional study, the use of U-Nets trained with region-specific context was investigated for accurately delineating the boundaries of different rectal structures (lumen, outer rectal wall, and perirectal fat) on post-nCRT T2w rectal MRI. Notably, across multiple external validation cohorts, region-specific U-Nets were found to be comparable to the inter-reader agreement between two radiologists as well as yielding significantly more accurate delineations compared to a traditional multi-class U-Net approach.

The observation that region-specific U-Nets outperform a traditional multiclass U-Net is consistent with previous work from outside the rectal imaging domain. For example, CNN models have been evaluated for individually segmenting sub-regions in brain tumors (the whole tumor region, the tumor core region, and the active tumor region) as well as in prostate cancer (whole prostate gland, the central gland, and the peripheral zone) on T2w MRI scans (19, 20). These studies found models developed specifically for each sub-region yielded more accurate segmentations (ranging from 75 to 92% overlap with expert annotations), While these findings resonate the results presented in this study (85–95% overlap for region-specific outer rectal wall and lumen U-Net), this study further demonstrates that training U-Nets with region-specific context can yield performances comparable to the agreement between multiple readers. The latter finding is an important prerequisite to automating the laborious manual annotation process that is typically utilized in image analytics studies.

This study represents one of the first efforts to examine automated segmentation of multiple anatomic rectal structures (outer rectal wall, lumen, and perirectal fat) on post-nCRT T2w MRI scans from rectal cancer patients. While a majority of previous studies have focused on automated delineation of rectal tumor extent on pre-nCRT MRI (12, 29–31), these have largely involved comparison against annotations from a single reader using data from a single institution. The most closely related studies leveraged U-Nets for segmenting the entire rectum alone on pre-nCRT, T2w MRI (14, 17). While these studies reported cross-validated DSC scores of 90–93% overlap with expert annotations, they primarily involved a single institution (with no external validation cohort) and only delineated the entire rectum as a single region (i.e., the outer rectal wall and lumen were considered a single structure). This work builds and expands on these attempts by developing region-specific U-Nets for accurately delineating multiple rectal structures using a multi-institutional cohort of post-nCRT T2w MRI scans (including external validation), which are known to be more visually confounded and harder to interpret than pre-nCRT imaging due to the presence of treatment effects such as fibrosis and edema (6, 32). The excellent performance achieved by using U-Nets trained with region-specific context was likely a result of these models being able to capture detailed aspects of anatomic boundaries more accurately for regions of different shapes and sizes such as between outer rectal wall, lumen, and perirectal fat on post-treatment MRI.

The generalizability of the wall and lumen-specific U-Nets in the current study was highlighted by their strong performance in holdout testing on patients whose imaging characteristics were different from that of the training cohort (e.g., coronal MRI, without rectal gel, or visibly poor image quality) as well as on patients from an external institution, respectively. The region-specific U-Nets were able to accurately identify the outer rectal wall and lumen even when the appearance of the MRI scan was significantly different (lumen boundaries are more obscured when rectal gel is not used), noisier, or acquired in a different acquisition plane (coronal vs. axial). Region-specific U-Net models were found to be generalizable likely because they were optimized to identify features unique to each anatomic region (both locally as well as semantically) in addition to the use of image augmentation approaches. Of the three imaging characteristics explored, the region-specific U-Nets yielded the lowest performance on MR images of poor quality (despite applications of multiple corrections including resampling, correcting for bias field, and standardizing pixel intensities), indicating the significant role image quality can play in downstream analytical image tasks (33). Additionally, of the three region-specific U-Nets, the fat-specific model yielded the most variable performance. The inconsistent performance of the fat-specific U-Net likely stems from the varied distribution of the perirectal fat throughout the rectum (34). Perirectal fat is fairly apparent in the upper and mid-rectum (especially in relation to the distinct mesorectal fascia boundaries), compared to the lower rectum (where puborectalis sling, levator ani, and sphincter muscles confound the fat boundary) (32). The fat-specific U-Net may thus require additional optimization to delineate the perirectal fat in the lower rectum.

Limitations to the present study can be acknowledged. While the cohort was moderately sized (*N* > 90 patients), this study included multiple independent holdout testing cohorts to comprehensively demonstrate the robustness of region-specific U-Nets in different settings (imaging differences, external validation) as well as comparison against multiple readers. Post-processed T2w MRI scans were utilized in this study when developing and validating the automated segmentation model, rather than specifically evaluating the impact of each of these processing steps individually on the U-Net models. The U-Net models were also developed as 2D architectures as opposed to 3D, primarily to ensure sufficient data was available for region-specific optimization. However, all 2D segmentations were evaluated on a 3D pseudo-volumetric basis to examine how well region-specific U-Net segmentations overlapped with reader annotations.

## 5. Conclusion and future work

In conclusion, this study represents the first multi-institution, multi-reader study to investigate U-Net models with region-specific context for annotation of lumen, outer rectal wall, and perirectal fat on post-treatment T2w MRIs. Results presented here demonstrated that U-Nets trained with region-specific context, as opposed to a multiclass U-Net, are better optimized to learn the confounded boundaries of different rectal tissue regions on post-treatment MRI. Automated segmentation of rectal structures on post-treatment T2w imaging is a key step toward improved quantitative evaluation of tumor extent *in vivo* as well as building more accurate downstream computational analytic tools for rectal cancers (35). Future work will investigate 3D region-specific U-Nets, automated identification of tumor regions both before and after chemoradiation, and to determine which image processing operations are most critical for image-based phenotyping in rectal cancers.

## Data availability statement

The data analyzed in this study is subject to the following licenses/restrictions: The datasets utilized in this study may be made available upon reasonable request and pursuant to relevant data-use agreements. Requests to access these datasets should be directed to SV, sev21@case.edu.

## Author contributions

SV guarantor of this manuscript, conceived and supervised the project, designed the experiments, oversaw the analysis, and as well as wrote and edited the final manuscript. TD performed data preparation/processing, data analysis, figures visualizations, and manuscript writing. JA, KB, PC, and HL assisted with data preparation/processing, statistical analysis, and writing/editing of the manuscript. DL, SS, EM, WH, and AP performed detailed chart review (including follow-up) and provided access to MRI scans and clinical variables. RP, JG, and AP provided annotations of MRI scans. KB assisted in obtaining and annotating MRI scans under the supervision of RP. DL, RP, JG, and AP provided clinical input on experimental design and assistance in editing the manuscript. All authors approved the final version of the article.
