# Supplementary material for: Region-specific deep learning models for accurate segmentation of rectal structures on post-chemoradiation T2w MRI: a multi-institutional, multi-reader study
Source: Front Med (Lausanne). 2023 May 11;10:1149056. doi: 10.3389/fmed.2023.1149056 (PMC10213753; doi:10.3389/fmed.2023.1149056)
Supplement: Supplementary file 1 [file Data_Sheet_1.pdf]

Supplementary Material

Region-specific deep learning models for accurate segmentation of rectal structures on post-chemoradiation T2w MRI: a multi-institutional, multi-reader study

Thomas DeSilvio, Jacob T. Antunes, Kaustav Bera, Prathyush Chirra, Hoa Le, David Liska, Sharon L. Stein, Eric Marderstein, William Hall, Rajmohan Paspulati, Jayakrishna Gollamudi, Andrei S. Purysko, Satish E. Viswanath*

*** Correspondence:** Satish E. Viswanath: satish.viswanath@case.edu

1 Supplementary Figures and Tables

1.1 Supplementary Figures

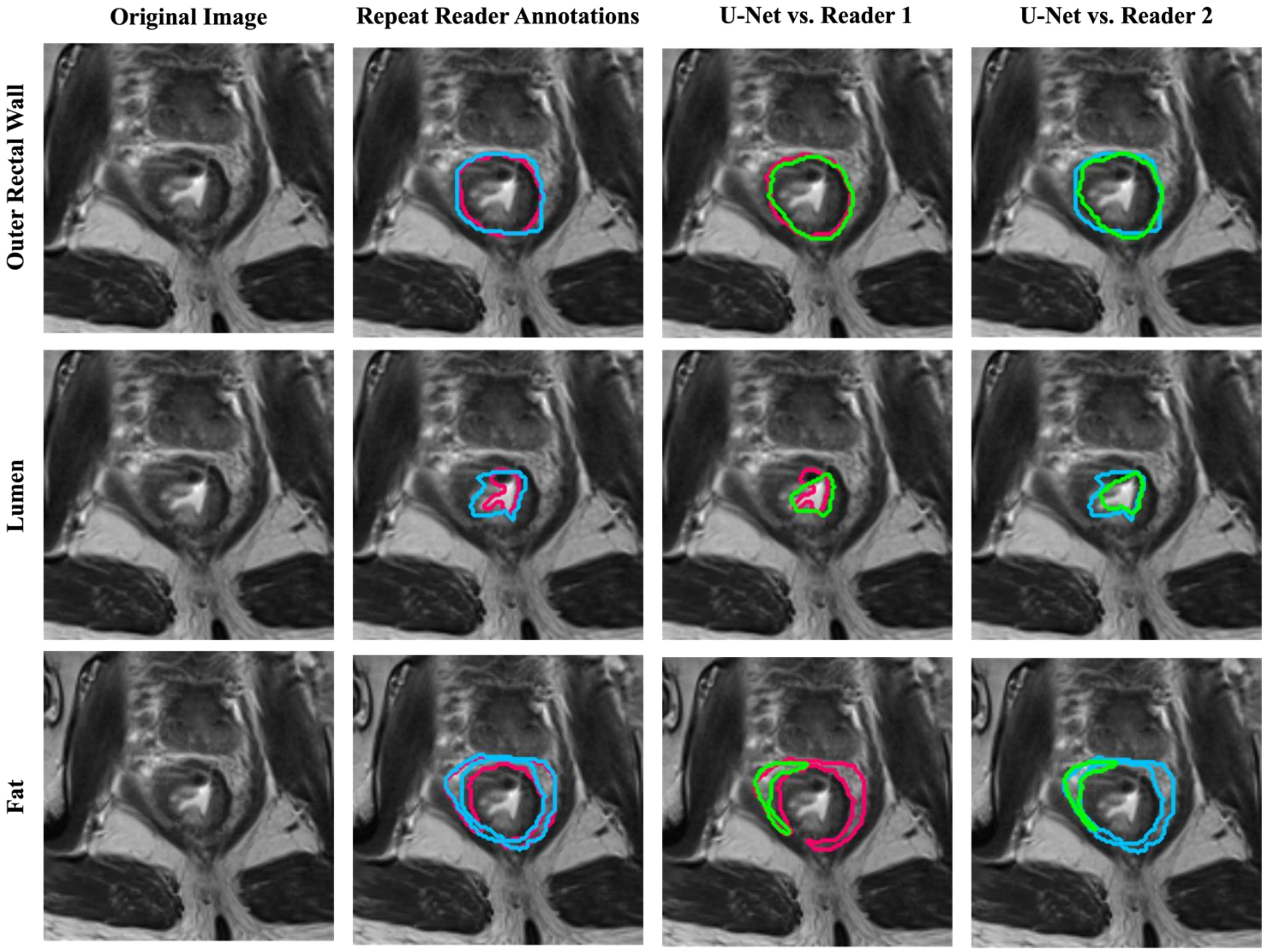

Figure S1: Representative segmentations of each region generated by Multiclass U-Net (green) compared to annotations of reader 1 (red) and reader 2 (blue) on a single patient in holdout testing sub-cohort C1.

1.2 Supplementary Tables

Table S1: Summary of previous deep learning approaches for segmenting rectal structures on T_2-weighted MRI. Note that a majority are single-institutional and only leverage pre-treatment MRI, while largely focusing on the tumor alone.

Authors	Time of MRI Scan	Number of Studies	Model & Approach	Number of Institutions	Number of Readers	ROIs Delineated	DSC	Year
Jian, et al.[12]	pre-nCRT	512	- 2D VGG-16<br>- 5 slices/study<br>- Cross-validation only	1	1	Tumor	0.830	2018
Wang, et al.[13]	pre-nCRT	93	- 2D U-Net<br>- Cross-validation only	1	2	Tumor	0.910	2018
Kim, et al.[14]	pre-nCRT	133 (cross-validation only)	- 2D U-Net<br>- 2-3 slices/study,<br>- Cross-validation only	1	1	Tumor, Rectal Wall	0.810, 0.900	2019
Lee, et al.[15]	pre-nCRT	457	- 2D VGG-16<br>- 2 slices/study,<br>- Cross-validation only	1	1	Tumor, Rectal Wall	0.742, 0.943	2019
Knuth, et al.[16]	pre-nCRT	192	- 2D U-Net<br>- 7-8 slices/study<br>- 1 validation cohort	2	2	Tumor	0.770	2022
Hamabe, et al.[17]	pre-nCRT	201	- 3D U-Net<br>- Cross-validation only	1	1	Tumor, Rectal Wall, Perirectal Fat	0.727, 0.930, 0.917	2022
**Current study**	**post-nCRT**	**92**	**- 2D region-specific U-nets<br>- 3 validation cohorts**	**3**	**2**	**Outer Rectal Wall, Lumen, Perirectal Fat**	**0.922, 0.897, 0.713**	**2023**

Table S2: Inter-reader agreement (reader 1 vs. reader 2) and performance of multiclass U-Net on holdout testing sub-cohort C1.

Region	Comparison	Median Dice	Median Hausdorff Distance	Median Fréchet Distance
Outer Rectal Wall	Reader 1 vs. Reader 2	0.946 ± 0.042	2.65 ± 0.584	2.83 ± 0.645
	Multiclass U-Net vs. Reader 1	0.787 ± 0.193*	3.00 ± 0.830*	3.16 ± 0.855*
	Multiclass U-Net vs. Reader 2	0.833 ± 0.191*	3.00 ± 0.833*	3.16 ± 0.849*
Lumen	Reader 1 vs. Reader 2	0.873 ± 0.199	2.45 ± 0.601	2.83 ± 0.613
	Multiclass U-Net vs. Reader 1	0.741 ± 0.293*	2.83 ± 0.955*	3.00 ± 0.933*
	Multiclass U-Net vs. Reader 2	0.768 ± 0.285*	2.65 ± 0.760*	3.00 ± 0.784*
Fat	Reader 1 vs. Reader 2	0.866 ± 0.261	3.16 ± 0.847	3.46 ± 0.947
	Multiclass U-Net vs. Reader 1	0.695 ± 0.368*	3.87 ± 1.12*	4.24 ± 1.18*
	Multiclass U-Net vs. Reader 2	0.746 ± 0.362*	3.87 ± 1.01*	4.24 ± 1.05*

* Denotes p-values < 0.008. p-Values were calculated by Wilcoxon Ranksum.
